# Platelet generation in vivo and in vitro

**DOI:** 10.1186/s40064-016-2384-1

**Published:** 2016-06-21

**Authors:** Biao Wang, Jiansheng Zheng

**Affiliations:** Department of Burns and Plastic Surgery, The 175th Hospital of PLA, Affiliated Southeast Hospital of Xiamen University, Zhangzhou, 363000 Fujian China

**Keywords:** Platelet manufacture, Adipose-derived stem cell, Physiologic thrombopoiesis

## Abstract

Platelet (PLT) transfusion, which is the primary cell therapy for thrombocytopenia, has been a source of concern in recent years due to its limitations of donor-dependent supply and soaring costs. In vitro platelet generation on an industrial scale is a possible solution requiring exploration. The technology of platelet generation ex vivo has been widely studied across the world, though the mechanisms of physiological thrombopoiesis and platelet biology function in vivo still remain elusive today. Various culture systems have been studied, most of which proved quite inefficient in generating functional platelets ex vivo, so there is still a long way to reach our ultimate goal of generating a fully functional platelet in vitro on an industrial scale. This review integrates the latest research into physiological platelet biogenesis and ex vivo-platelet/megakaryocyte (MK) generation protocols with a focus on the ability to generate PLT/MK in large quantities, summarizes current culture systems based on induced human pluripotent stem cells and adipose-derived stem cells, and discusses significant challenges that must be overcome for these approaches to be perfected.

## Background

In healthy adults, human platelets maintain a normal level of approximately (150–400) × 10^3^/μl in blood, with a short life span of only 7–10 days (Lu et al. 2011). There is considerable evidence that platelets play an essential role in hemostasis and thrombosis (Linden and Jackson 2010), maintaining vascular integrity (Ho-Tin-noé et al. 2011), angiogenesis, innate immunity (Semple et al. 2011), inflammation, cancer biology (Gay and Felding-Habermann 2011), and wound healing, among others (Menter et al. 2014). Clinically, abnormal changes in platelet quality and/or quantity will occur in response to various life-threatening conditions, including thrombocytopenia, thrombocythemia, idiopathic thrombocytopenic purpura, myelodysplastic syndromes (MDS), chemotherapy-induced thrombocytopenia, aplastic anemia, human immunodeficiency virus (HIV) infection, and major cardiac surgery (Thon and Italiano 2010). To mitigate risks associated with these conditions, platelet transfusion is most commonly used as a kind of cell therapy. However, platelet transfusions are limited by alloimmunization, availability, and expense. Transfusions are, at present, totally donor dependent, which leads to a limited supply for clinical therapy and creates a constant shortage in transfusion supplies. Meanwhile, the steady increase in demand is challenging every blood station around the world. Fortunately, there are considerable studies providing abundant evidence that functional platelets can be generated in vitro, which shows significant advantages over the donor-dependent supply, though many aspects of the process are poorly understood. The technology to generate fully functional platelets in vitro on an industrial scale for clinical platelet transfusion is needed urgently (Reems et al. 2010).

## Physiological thrombopoiesis process

### Hematopoiesis and the marrow microenvironment

We consider that the ideal culture system to manufacture platelets in vitro should be characterized by certain principles, including easiest operating system, most abundant precursor cell source, shortest culture period, highest yield efficiency, and fully functional platelets (EASHF rule). To most closely mimic the in vivo process of megakaryocyte (MK)/platelet (PLT) development when developing in vitro approaches to platelet generation, it is necessary to briefly review the in vivo process primarily responsible for megakaryocyte development and platelet biogenesis.

Currently accepted theory is that platelets are formed and released into the bloodstream by precursor cells called megakaryocytes derived from pluripotent hematopoietic stem cells (HSCs) that reside within the bone marrow. For additional information regarding this topic please see review Yin and Li (2006). Bone marrow (BM) consists of a hematopoietic component and surrounding tissue. Mesenchymal stem cells (MSCs) give rise to a variety of cell types, including myocytes, adipocytes, fibroblasts, endothelial cells, and osteoblasts (Taichman 2005). There are at least two niches in the bone marrow having been identified for HSC hematopoiesis, including the osteoblastic niche and the vascular niche.

Schofield first advanced the concept of niches in 1978, describing the microenvironment where stem cells reside in bone cavity (Schofield 1977). The bone cavity is filled with soft BM and other components. The stem cell niche is composed of a variety of cells for the maintenance of stem cells and generates extrinsic factors that control stem cell number (Li and Xie 2005) and fate with regard to self-renewal versus differentiation (Fuchs et al. 2004; Spradling et al. 2001). In mammals, some hematopoietic cells live next to the endosteal bone surface, which is lined mainly by osteoblasts, suggesting reciprocal communication between the two cell types. Osteoblasts have been identified as responsible for generation of a variety of hematopoietic growth factors (Taichman et al. 1996, 2001). Some studies demonstrated that osteoblasts were key components of the HSC niche for supporting HSC growth (Zhang et al. 2003; Calvi et al. 2003; Visnjic et al. 2004). Additionally, experiments on osteoblast depletion and ablation of endothelial cells showed hematopoietic failure (Avecilla et al. 2003). Another study further demonstrated adhesion of HSCs to osteoblasts on the endosteal surface, an essential molecular mechanism for maintaining HSC in quiescence (Arai et al. 2004). The vascular niche consists of hematopoietic cells and endothelial cells, similarly to the composition of the osteoblastic niche; the two niches are also closely related during development. The vascular niche and the osteoblastic niche are both derived from hemangioblasts (Kopp et al. 2005). Functionally, vascular endothelial cells maintain HSCs in vitro and are required for hematopoiesis in vivo (Li et al. 2004). Studies have suggested that the vascular niche is the site of HSC differentiation and mobilization (Avecilla et al. 2003; Kopp et al. 2005). Thus, it is plausible that the osteoblastic niche provides a quiescent environment for HSC maintenance and the vascular niche offers an alternative niche for mobilized stem cells and promotes proliferation and further differentiation or maturation into the circulation system. There are numerous publications indicating that HSC return to the BM, so-called homing. However, the underlying physiological function of these events remains elusive (Wright et al. 2001).

In summary, both the osteoblastic niche and the vascular niche have a likely important role in HSC mobilization, which consists of HSCs leaving the osteoblastic niche, mobilizing to the vascular niche, entering blood vessels, and circulating in the vascular system. Homing of stem cells is simply the reversal of this process (Lapidot et al. 2005).

### Megakaryopoiesis

A significant proportion of studies result in the conclusion that the global process of thrombopoiesis includes: megakaryocyte precursor development from stem cells and cell fate determination; endomitosis and the development of polyploidy; internal membrane, tubular, cytoskeletal, and granule evolution directed by key transcription factors and transcriptional programs; regulated apoptosis; and additional intricate and time-dependent events (Gordge 2005; Italiano et al. 2007).

The megakaryocyte is the central cell in thrombopoiesis. Megakaryocytes originate from HSCs like other terminally differentiated hematopoietic cells, such as erythrocytes and neutrophils (Ogawa 1993). Further, Najet Debili and his colleagues further prove that megakaryocytes and erythroid lineages arise from a common megakaryocyte-erythroid progenitor (MEP) derived from the common myeloid progenitor (Debili et al. 1996), yet the signals that regulate the final separation of these lineages are not well understood. Megakaryopoiesis is established first in the embryonic yolk sac, though studies have shown that platelets are not very important for fetus survival (Shivdasani et al. 1995). Broadly speaking, there may be four primarily sequential biological stages for thrombopoiesis and megakaryopoiesis. The self-renewal of HSCs living in the BM osteoblastic niches is the first step. Subsequently, stem cells proliferate and differentiate to increase the number of MK progenitors. In the third step of maturation events, MK progenitors undergo a process known as endomitosis that the DNA continue to replicate, but cytokinesis and nuclear division fails and, as a result, they require a DNA content of up to many times the normal complement of 46 chromosomes (i.e., >2 N). However, a significant proportion of MKs containing two physically separated nuclei have been reported recently. The fourth and last stage is characterized by platelet assembly and release (Pang et al. 2005; Reems et al. 2010). Thus, it is easy to understand that more MKs make more platelets in this process. To date, large quantities of transcription factors and cytokines involving megakaryopoiesis, including granulocyte macrophage colony-stimulating factor (GM-CSF), IL-3, IL-6, IL-11, IL-12 (Gordon and Hoffman 1992), IL-1α and leukemia inhibitory factor (LIF) (Gordon and Hoffman 1992; Vainchenker et al. 1995), et al. The most noteworthy is that thrombopoietin (TPO), which is synthesized predominantly in the liver (Jelkmann 2001), has been identified as markedly stimulating megakaryoctye production by combining with the MPL receptors (Kaushansky et al. 1994; Lok et al. 1994). In the end, the senescent MK nucleus remaining after platelet release is, predictably, disposed of by apoptosis and phagocytosis (Gordge 2005).

### Proplatelet generation and platelet release

Platelet production is recognized as the final stage of megakaryoctye development so far. Mature megakatyoctyes have the ability to shed 2000–10,000 individual platelets, which shows great clinical significance. Most observers pay much attention to the mechanisms active in the cytoskeleton of proplatelets and platelets (Hartwig and Italiano 2006; Thon et al. 2010). The current model of platelet formation recognizes that mature megakaryocytes extend long, branching processes, called proplatelets, which are composed of platelet-sized swellings in tandem arrays that are connected by thin cytoplasmic bridges (Italiano et al. 1999). Proplatelets have been identified both in vitro and in vivo (Leven 1987; Tablin et al. 1990), and proplatelet-producing megakaryocytes yield platelets that are parallel to blood platelets in structure and function (Behnke 1969; Choi et al. 1995). Nevertheless, the final stages of proplatelet maturation and platelet release remain little understood. Recently, Jonathan N. Thon and his colleagues identified a new intermediate stage in platelet production with enriched proplatelet populations characterized for presence/absence of a nucleus, morphology, and size after double staining for nuclei and microtubules, termed preplatelets. According to their survey, preplatelets are anucleate discoid particles dramatically larger (2–10 μm) than blood platelets and have the ability to reversibly convert into proplatelets during cell culture (Thon et al. 2010).

Platelet production begins with the erosion of one pole of the megakaryocyte to generate large pseudopodial-like structures that elongate, thin, and branch to yield slender tubular projections of uniform diameter (2–4 μm). Tablin and Leven first gained insight into the mechanics of platelet formation, showing proplatelet elongation to be dependent on microtubules using specific poisons of microtubule assembly (Tablin et al. 1990). We have learned that platelets mature only at the ends of highly branched proplatelets, and individual organelles are sent from the megakaryocyte cell body to the proplatelet where they move bidirectionally until they are captured at the proplatelet ends (Richardson et al. 2005). The sliding movement of thick bundles of microtubules contributes to proplatelet formation, and by passing one another within the proplatelet shafts it is the primary motor for proplatelet elongation. The slide rate is expected to be 4–5 μm/min within the proplatelets; the underlying mechanism remains unclear (Hartwig and Italiano 2006). Continued bidirectional polymerization of microtubules at each end of the barbell proplatelet forms two well-defined platelet-sized microtubule loops at each end, and two individual platelets remain after a fission event.

**Fig. 1:**
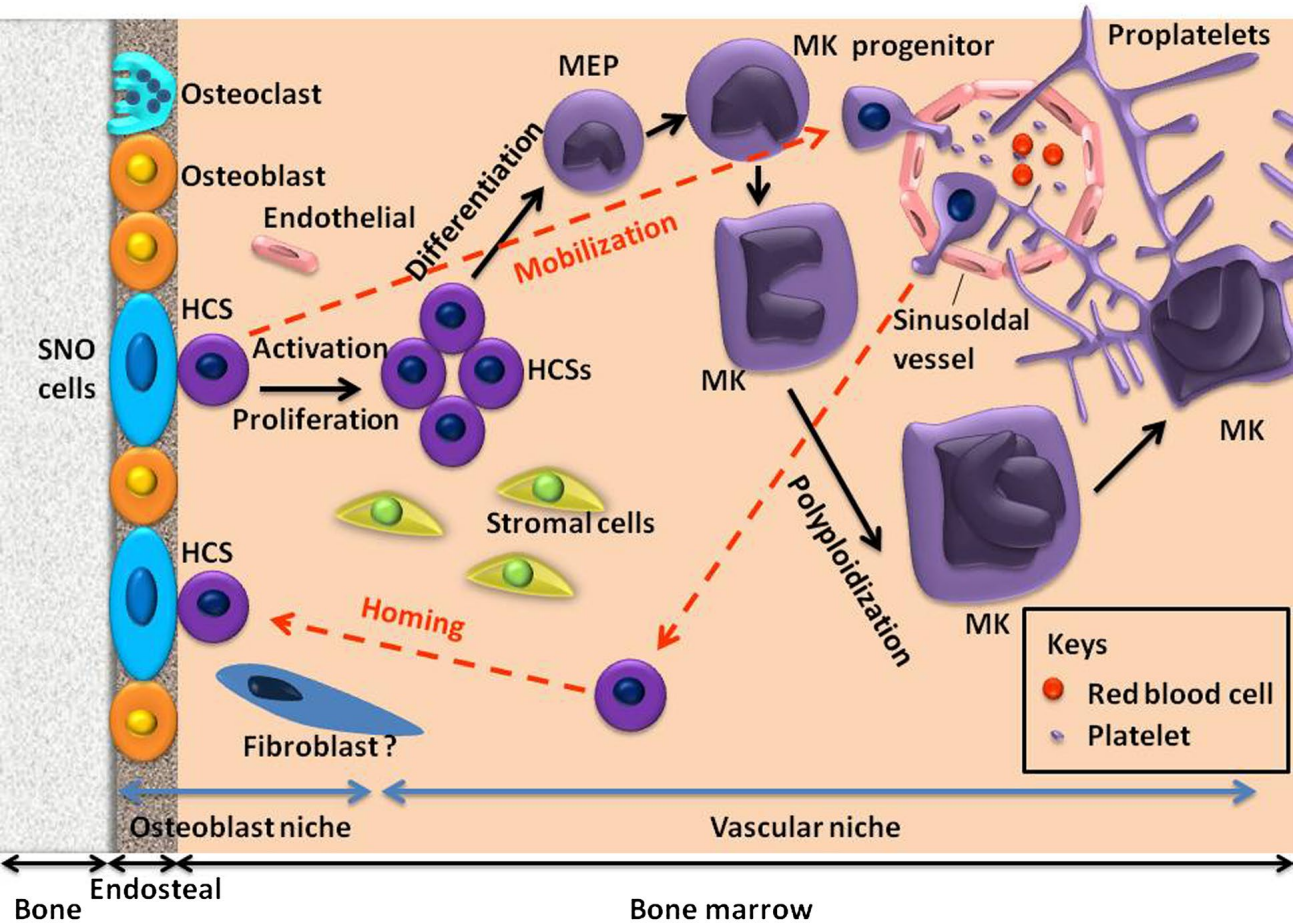
The process of megakaryocyte differentiation and platelet production from HSCs within the niches. There are at least two niches in the bone marrow (BM) identified for HSC hematopoiesis, including the osteoblastic niche and the vascular niche. The HSCs live next to the endosteal bone surface, lined mainly by osteoblasts, which constitute the physiological microenvironment called the osteoblastic niche, providing a quiescent environment for HSC maintenance. The vascular niche consists of HSCs and endothelial cells, offers an alternative niche for mobilized stem cells, and promotes proliferation and further differentiation or maturation into the circulation system. There may be four primarily sequential biological stages for thrombopoiesis and megakaryopoiesis. The self-renewal of HSCs living in the BM osteoblastic niches is the first step. Stem cells then proliferate and differentiate to increase the number of MK progenitors. In the third step of maturation events, MK progenitors undergo a process known as endomitosis. The fourth stage is characterized by platelet assembly and release. The current model of platelet formation recognizes that mature megakaryocytes extend long, branching processes, called proplatelets, which are composed of platelet-sized swellings in tandem arrays that are connected by thin cytoplasmic bridges. Platelet production begins with the erosion of one pole of the megakaryocyte to generate large pseudopodial-like structures that elongate, thin, and branch to yield slender tubular projections of uniform diameter (2–4 μm). The product released by megakaryocytes may be proplatelets, and the product released by proplatelets may be preplatelets and/or platelets of various sizes. The *red dotted line* showed HSC circulation, which contains HSCs leaving the BM, entering the vascular system (termed *mobilization*), and returning to the BM (known as *homing*). However, the underlying physiological function of these events remains elusive. *SNO cell* spindle-shaped N-cadheri^+^CD45^−^ osteoblastic cell, *MEP* megakaryocyte-erythroid progenitor, *HSCs* hematopoietic stem cells, *MK* megakaryocyte

Underlying details of how mature platelets release from the proplatelet tips are still elusive. The product released by megakaryocytes may be proplatelets, and the product released by proplatelets may be preplatelets and/or platelets of various sizes. Whether the microtubule motors contribute to platelet release is unknown, but the fact that the dumbbell-shaped particles are easily released into the bathing media of megakaryocyte cultures may suggest that they are a preferred release form (Fig. 1).

### Flow shear force

Recent work shows that when mature human MKs are exposed to high shear forces proplatelet processes become apparent and platelets are released within 20 min. However, when left in static cultures often no platelets are generated or it takes several hours for platelets to be released (Dunois-Lardé et al. 2009). Shear enhances platelet release from proplatelet tips in marrow sinusoids in vivo on the basis of intravital microscopic studies of mice (Junt et al. 2007). Whether the flow shear stress is the only mechanical force to sever platelets from proplatelet/preplatelet processes requires an intricate series of experiments to research, but the static nature of the majority of current culture protocols leads to low yields of platelets from culture-derived MKs. Studies of thrombopoiesis in lungs are consistent with shear force’s function in platelet release (Kaufman et al. 1965; Schwertz and Weyrich 2010). Many studies indicate that the lung is a site of platelet biogenesis, and in vitro modeling demonstrates that central events in thrombopoiesis are directed by cell-autonomous mechanisms and do not require the specific BM environment (Italiano et al. 2007). Proplatelet numbers were higher in prepulmonary blood than on the systemic side of lungs in rats, whereas platelets numbers were higher in systemic blood, which suggests that pulmonary vessels may be providing particularly favorable rheological conditions for the fission process (Kaufman et al. 1965; Handagama et al. 1987).

In summary, the HSC circulation involves mobilization, HSCs leaving the BM, entering the vascular system, and homing, i.e., returning to the BM. The main process of thrombopoiesis consists of: megakaryocyte precursor development from stem cell and cell fate determination; endomitosis and the development of polyploidy; internal membrane, tubular, cytoskeletal, and granule evolution directed by key transcription factors and transcription programs; regulated apoptosis (Weyrich and Zimmerman 2013); proplatelet formation; final platelet release by flow shear forces and other mechanisms; other intricate processes, etc. Nevertheless, little is known about physiological megakaryopoiesis and thrombopoiesis processes to date, and further knowledge is needed urgently.

## Platelet generation in vitro

Since the first report by Choi et al. (1995) that human megakaryocytes and platelets could be generated in vitro from CD34^+^ peripheral blood progenitor cell, multiple researchers also have proved that the generation of megakaryocytes and platelets could start with CD34^+^ progenitor cells derived either from umbilical cord blood (Tao et al. 1999), fetal liver (Ma et al. 2000), peripheral blood (Bruyn et al. 2005), human embryonic stem cells (hESCs) (Gaur et al. 2006; Takayama et al. 2008), human induced pluripotent stem cells (hiPSCs) (Gekas and Graf 2010; Takahashi et al. 2007), or bone marrow (Guerriero et al. 1995). Multiple culture systems appear, though each system has its own different limitation for large scale of regeneration of megakaryocytes and platelets. Most of these progenitor cell sources still require a continuous supply of donors due to limited expansion potential, however, iPSCs and hESCs show great capacity to serve as renewable and unlimited sources of cells that can be expanded in culture and differentiated into megakaryocytes (Lambert et al. 2013). Nevertheless, there remains a concern that any cellular product derived from pluripotent ESCs or iPSCs could be oncogenic or teratogenic (Ben-David and Benvenisty 2011). Recently, Wang et al. (2012) first produced functional platelets using human endometrial stromal stem cells (hESSCs) in a serum-free medium supplemented with TPO in an in vitro culture system. However, there is still no one universally accepted perfect stem cell source for in vitro MK/PLT production.

### Human induced pluripotent stem cells (iPSCs)

Kazutoshi Takahashi and Shinya Yamanaka first generated induced pluripotent stem cells (iPSCs) directly from a mouse embryonic or adult fibroblast culture by introducing four essential transcription factors: Oct3/4, Sox2, c-Myc, and Klf4 (Takahashi and Yamanaka 2006). In this study, their operation of subcutaneous injection of iPSCs into nude mice, resulting in tumors containing varieties of tissue from all three germ layers (Takahashi and Yamanaka 2006), which shows the promising capacity of iPSCs in differentiation. Subsequently, Takayama et al. (2010) successfully produced platelets from individual four-factor hiPSC clones established from human dermal fibroblasts (HDFs)These human iPSC-derived platelets show satisfactory functionality in vitro and in vivo in the NOG (nod-scid/IL-2 γc-null) mouse thrombocytopenia model; however, large scale platelet production is little mentioned in this article. In addition, they also provide insight into the role of c-Myc in megakaryopoiesis and thrombopoiesis (Takayama et al. 2010). The assumption that c-Myc has both positive and negative targets that influence megakaryopoiesis and that these targets are regulated in a manner sensitive to different transcription factor concentrations is accepted now Gekas and Graf (2010). Similarly, Nishimura et al. (2013) generated for the first time MKs and functional platelets in vitro using canine induced pluripotent stem cells (ciPSCs) with the 29 % ratio of peripheral platelets positive for CD41/61 Abs and 17 % of platelets from culture supernatant (Nishimura et al. 2013), which shows the possibility of MK generation and the release of functional platelets from iPSCs.

Little research about large-scale megakaryopoiesis and thrombopoiesis from iPSCs was reported until recent years. In the US, Feng et al. (2014) generated universal platelets from hiPSCs in the absence of serum and animal feeders, which is a simplified culture system following the EASHF rules, in less than 20 days. In total they generated 2.06 × 10^9^ megakaryocyte progenitors (MKPs) from 1.26 × 10^8^ iPSCs, an average of >16 MKPs per single iPSC input. Significantly, they deleted the β2-microglobulin gene, generating “universal platelets” that are negative for major histocompatibility antigens (Feng et al. 2014). They also demonstrated that approximately 30 platelets per iPSC MK could be generated in vitro in the presence of shear force not too long ago (Thon et al. 2014); however, efficiency is currently still low compared to >2000 platelets/MK in BM. Recently, Nakamura et al. (2014) sought to establish stable immortalized megakaryocyte progenitor cell lines (imMKCLs) for clinically applicable generation of platelets from iPSCs. However, the technology of manufacturing functional platelets ex vivo from iPSCs is a relatively sophisticated reprogramming method. Thus, the challenge for reaching industrial scale generation of ex vivo platelets is still present. We propose that simpler culture systems and less strict experiment protocols for platelets generation from iPSCs are needed to take the next step forward.

### Adipose-derived stem cell (ADSC)

Compared with reprogrammed iPSCs, the advantage of using subcutaneous adipose tissues for platelet production is obvious, since subcutaneous adipose tissues are easily obtained and available in quantity. Japanese researchers Yumiko Matsubara et al. (2009) generated megakaryocyte and functional platelets from subcutaneous adipocyte precursor cells in an in vitro culture system for the first time in 2009.

Briefly, they used a two-step procedure in their platelet generation experiment. First they cultured primary human adipocyte precursors using Preadipocyte Growth Medium-2 BulletKit™ Medium, designated “mature adipocyte media,” for differentiation into mature adipocytes over 12 days. By day 12, approximately 80 % of cells showed differentiation into mature adipocytes. Secondly, cells were cultured in conditioned media in the presence of thrombopoietin (TPO), designated “TPO media,” to further differentiate into MK lineages for another 12 days. As a result, approximately 2 × 10^6^ ± 2500 and 15 × 10^4^ ± 270 of MKs and platelets were produced, respectively. The DNA ploidy of the adipocyte precursor cell-derived MKs reached 16 N (Matsubara et al. 2009). As stated previously, platelet production in vivo is a highly efficient process, with 2000–10,000 platelets being produced from each MK precursors cell (Kaufman et al. 1965; Long 1998). However, many aspects remains imperfect in the experiment, such as the failure to identify the cell population that is responsible for generation of MKs and platelets, though the authors conjecture that the cell population differentiated into mature adipocytes may be responsible for megakaryopoiesis and thrombopoiesis. They state their assumption that mature adipocytes have the ability to differentiate into MKs and platelets, but have no experiments proving it. As for the functional assay of adipocyte precursor cultured cell-derived MKs/PLTs, they performed it only ex vivo; performance in vivo is blank. Regardless, they have opened promising prospects for platelet generation ex vivo in large quantities, since subcutaneous adipose tissue is easily obtained and available in large quantities (Matsubara et al. 2012) and provides an abundant and accessible source of preadipocytes for regeneration medicine.

Additionally, the mouse bone marrow stroma cell line OP9 is regarded as pre-adipocyte and has been utilized as feeder cells for the differentiation of stem cells into MK lineages. Matsubara et al. successfully generated functional MKs and PLTs from OP9 cells using MK lineage induction medium. Moreover, they took a further step forward to explore the mechanism of MK differentiation from pre-adipocyte OP9 cells. They revealed that the process of MK differentiation from OP9 cells had much to do with the gene expressions of p45NF-E2, FOG, Fli1, GATA2, RUNX1, TPO, and c-MPL, which regulates megakaryopoiesis and thrombopoiesis. Among them, the p45NF-E2 expression they observed was increased most markedly during the differentiation of OP9 cells into MK lineages. They further studied the effect of OP9 cells transfected with p45NF-E2 on MK and PLT production and found that the gene p45NF-E2 significantly elevated 1 × 10^6^ OP9 cells, derived MKs production from (2.2 ± 1.6) × 10^4^ for 1 × 10^6^ empty-vector-OP9 cells to (3.3 ± 1.8) × 10^4^ for p45NF-E2-OP9 cells, respectively, and the number of platelets generated from 1 × 10^6^ OP9 cells was (2.9 ± 2.6) × 10^5^ for the p45NF-E2-OP9-derived platelets and (1.0 ± 7.3) × 10^5^ for the empty-vector-OP9-derived platelets, which indicated that p45NF-E2 has a vital role in the generation of MKs and platelets from OP9 cells, the pre-adipocytes (Matsubara et al. 2013).

### Human embryonic stem cells (hESCs)

Eto et al. (2002) successfully generated large, polyploidy MKs that produced proplatelets cocultured with OP9 stromal cells in the presence of thrombopoietin, IL-6, and IL-11. In their system, IL-6 and IL-11 marginally enhanced the yield of viable, mature MKs, although they are not necessary for megakaryocytopoiesis. Typically, an initial culture of 1 × 10^4^ ES cells yields 6 × 10^4^ viable, αIIb-positive cells on day 12, of which two-thirds were large MKs. However, only 10 % of large MKs derived from ES cells were able to convert into proplatelets. Their study indirectly demonstrates that the efficient OP9 culture system can yield MKs on a sufficient scale and also indicates that MKs can be derived relatively rapidly and in quantity from ES cells. Eto et al. (2003) introduced detailed methods for generating megakaryocytes in quantity from murine ES cells. Similarly, other researchers Kitajima et al. (2003), Fujimoto et al. (2003), Vodyanik et al. (2005), Gaur et al. (2006) also successfully induced differentiation of human/murine ES cells into MKs utilizing the OP9 system, which convincingly demonstrates the abilities of the OP9 system in culturing MKs derived from ES cells. Further, Takayama et al. (2008) first found that vascular endothelial growth factor could promote the emergence of sac-like structures derived from embryonic stem cells (ES-sacs), which provided suitable conditions for hematopoietic progenitors. Their results also showed that relatively large numbers of mature megakaryocytes could be induced from hematopoietic progenitors within ES-sacs, which were then able to release functional platelets. And on average, (4.8 ± 0.2) × 10^6^ platelets were manufactured from initial 10^5^ hESCs in the presence of a combination of TPO, stem cell factor (SCF), and heparin, which was considered the lower limit of the yield (Takayama et al. 2008).

However, the problem of how to generate functional megakaryocytes and platelets from hESCs on a large scale remains effectively unsolved. Recently, Lu et al. (2011) took an important step toward generating an unlimited supply of platelets for transfusion. They first tried to utilize an efficient method to generate functional MKs from hESCs on a large scale with hemangioblasts/blast cells (BCs) instead of OP9 cells as intermediates. Although they indeed harvested 6 × 10^8^ CD41a^+^ MKs from about 1.0 × 10^7^ MA09 hESCs on a large scale as reported, on average only 6.7 ± 0.4 hESCs-PLTs were generated per hESCs-MK, the efficiency of which is still much lower than that observed in vivo as mentioned above. It is possible that the low efficiency is due to a shortage of necessary shear force and other biological cytokines yet unknown. Absolutely the culture system needs to be improved further. Another inevitable obstacle to massive utilization of hESCs in clinical use lies in human ethic and moral principles.

Of course, many other different kinds of cell sources have been found and cultured to generate MKs/PLTs in vitro, such as human embryonic stem cells (hESCs) (Olsen et al. 2006; Pick et al. 2013), hematopoietic stem cells (HSCs) (Debili et al. 1995; Mercher et al. 2008), induced megakaryocytes (iMKs) (Masuda et al. 2012), human CD34^+^ cells from bone marrow (BM), umbilical cord blood (CB) (Choi et al. 1995; Matsunaga et al. 2006), and even human endometrial stromal stem cells (hESSCs) (Wang et al. 2012). However, these cells cannot be utilized satisfactorily due to lack of resources and other reasons; therefore, it is not necessary to describe them in detail in the present article (Table 1). Exhilaratingly, Fuentes et al. (2010) offer an alternative approach to generating PLTs from megakaryocytes ex vivo. They infuse mature MKs directly into mice and platelets are formed and released in vivo with characteristics similar to those of normal platelets. According to their estimation, 100–200 platelets are generated per infused megakaryocyte (Fuentes et al. 2010). This approach relies on research showing that megakaryocytes release platelets in the lungs (Zucker-Franklin and Philipp 2000). Other reports (Kaufman et al. 1965; Lu et al. 2011) are consistent with these findings, including an Andrew S. Weyrich review of platelet physiological function in the lung (Kaufman et al. 1965; Weyrich and Zimmerman 2013) in 2013.

**Table 1:** Summary of different protocols in vitro for the efficient yield of PLTs/MKs and their functionality

Source of cells	Efficient yield of MKs	Efficient yield of PLTs	Platelet functionality	References
imMKCLs	No data	Three CD42b^+^ PLTs per Cl-2 imMKCL-MK; Ten PLTs per Cl-7 imMKCL-MK.	CD42b^+^; CD61^+^; PAR1^+^; CD49b^+^; CD29^+^ Positive integrin αIIβ3 activation and platelet aggregation. However, imMKCLs platelets gave less robust responses than fresh human platelets Thrombogenic activity in mouse models of thrombocytopenia	Nakamura et al. (2014)
iPSCs	No data	Average 9.8 × 10^5^ platelets were generated from 3 × 10^4^ ciPSCs.	The platelets were activated with ADP or thrombin and bound to fibrinogen The platelets had the same ultrastructures, OCS, and α-granules as peripheral platelets	Nishimura et al. (2013)
	A total of 2.06 × 10^9^ MKPs were generated from 1.26 × 10^8^ iPSCs, an average of > 16 MKPs per single iPSC input.	Approximately six platelets per MKP	iPSC platelets generated under completely serum- and feeder-free conditions are functional, forming platelet thrombi in vivo in macrophage-depleted NOD/SCID mice iPSC platelets circulated for at least 8 h in macrophage-depleted NOD/SCID mice	Feng et al. (2014)
ADSCs	Approximately 2 × 10^6^ MKs were gained from 10^7^ adipocyte precursor cells	15 × 10^4^ PLTs/10^7^ adipocyte precursor cells	adipocyte precursor cell-derived platelets are in response to thrombin CD41^+^; CD42a^+^; CD42b^+^ P-selection surface expression (+)	Matsubara et al. (2009)
	5 × 104 MKs from 10^6^ 3T3-L1 cells	1 × 10^5^ PLTs from 10^6^ 3T3-L1 cells	CD41^+^; CD42b^+^ The binding of fibrinogen to 3T3-L1-derived platelets was increased with stimulation by agonists	Matsubara et al. (2010)
	(3.3 ± 1.8) × 10^4^ MKs from 10^6^ p45NF-E2-OP9 cells	(2.9 ± 2.6) × 10^5^ PLTs from 10^6^ p45NF-E2-OP9 cells	fibrinogen binding(+) and P-selection surface exposure(+) CD41^+^; CD42b^+^	Matsubara et al. (2013)
hESSCs	5.5 × 10^4^ ± 500 MKs from 1.8 × 10^5^ ± 642 hESSCs	3 × 10^5^ ± 360 PLTs from 1.8 × 10^5^ ± 642 hESSCs	PLTs bind to fibrinogen and express CD62P after thrombin stimulation PLTs intracellular structures are similar to PLTs harvested from human peripheral blood	Wang et al. (2012)

Obviously it could be concluded from the table above that most of the culture systems that have been studied thus far are too inefficient to generate MKs/PLTs on a large scale. However, the advantage of using ADSCs for platelet production is obvious, since subcutaneous adipose tissues are easily obtained and available in quantity

*imMKCLs* immortalized megakaryocyte progenitor cell lines, *Cl-2* clone 2, *Cl-7* clone 7, *PAR1* protease-activated receptor 1, *ciPSCs* canine induced pluripotent stem cells, *OCS* open canalicular system, *MKP* megakaryocyte progenitor, *hESSCs* human endometrial stromal stem cells

All these cells have already proven capable of differentiating into megakaryocytes and platelets; however, for various reasons none is generally considered perfect or optimal for platelet generation on a large scale in vitro.

## Conclusion

In summary, to alleviate the platelet transfusion stress discussed above, researchers around the world have sought to generate platelets mainly in two directions. One direction is that platelets are generated in vitro from various cell sources, such as HSCs, ADSC, CB, hESCs, etc. The other direction is that mature megakaryocytes are generated ex vivo and then infused into experimental objects, based on the theory of megakaryocytes shedding platelets in lungs. In both directions one encounters many barriers. For instance, all ESCs and iPSCs are inherently tumorigenic and form teratomas in vivo, but differentiated platelet cultures for cell replacement therapy must be completely pure platelets without residual pluripotent cells. A generally accepted method for elimination of pluripotent cells is cell irradiation; however, as yet we have no idea whether the experimental object will have any adverse reactions when infused with large quantities of dead pluripotent cells fragments. BM and CB are donor dependent and the expansion capability of these cells is limited, and so on. The half-life of these platelets derived from infused MKs is slighter shorter than that of infused platelets, for reasons still unclear. In our opinion, ADSCs may play a starring role in industrial platelet generation due to abundant sources and differentiation potency. In addition, we propose that the perfect culture system to manufacture platelets in vitro should follow EASHF rules, including the easiest operation system, most abundant precursor cell source, shortest culture period, highest yield efficiency, and fully functional platelets. We believe that the dream of platelet generation in vitro on an industrial scale will come true 1 day by means of a multidisciplinary combination, like Biomaterials Science, Mechanical Physics, etc.
